# Rise and Fall of *Phytophthora infestans* Resistance to Non-Specific Fungicide in Experimental Populations

**DOI:** 10.3390/jof11090643

**Published:** 2025-08-30

**Authors:** Shao-Bin Fan, Meng Xie, Zu-Lei Xiang, Tong-Xin Xu, Wen-Jing Wang, Zong-Hua Wang, Hong-Li Hu, Li-Xia Chen, Li Tang, Jia-Sui Zhan, Li-Na Yang

**Affiliations:** 1 Key Laboratory of Biopesticide and Chemical Biology of Ministry of Education, College of Plant Protection, Fujian Agriculture and Forestry University, Fuzhou 350002, China; m13055257938@163.com (S.-B.F.);; 2 Fujian Key Laboratory on Conservation and Sustainable Utilization of Marine Biodiversity, Fuzhou Institute of Oceanography, Minjiang University, Fuzhou 350108, China; 3 College of Resources and Environment, Fujian Agriculture and Forestry University, Fuzhou 350002, China; 4 Zhengzhou Research Base, National Key Laboratory of Cotton Bio-Breeding and Integrated Utilization, School of Agricultural Sciences, Zhengzhou University, Zhengzhou 450001, China; 5 Department of Forest Mycology and Plant Pathology, Swedish University of Agricultural Sciences, SE-750 07 Uppsala, Sweden

**Keywords:** *Phytophthora infestans*, mancozeb resistance, non-specific fungicides, ABC transporters, endocytic proteins

## Abstract

**Background**: Fungicide resistance is one of the major factors threatening social and ecological sustainability. Many issues associated with the evolutionary processes and mechanisms of fungicide resistance in pathogens remain poorly understood, and better knowledge of these issues through experimentally observing the rise and fall of the resistance is critical for the development of effective management strategies to ensure food security and ecological health. **Methods**: An experimental evolution approach was used to continuously acclimate a series of *Phytophthora infestans* populations under different mancozeb conditions for 400 consecutive days. **Results**: We found that *P. infestans* developed mancozeb resistance after 200 days of acclimation. This resistance was associated with ABC transporters and endocytic proteins. Potentially due to fitness costs associated with aggressiveness, mancozeb resistance was reversible. And the pathogen exhibited comparable rates of resistance gain during acclimation and resistance loss during the reversal experiment. **Conclusions**: Our results suggest that this pathogen may also develop resistance to mancozeb. However, this type of resistance may not be persistent, implying the fungicides concerned could be reused in practice. These results provide new insights into the evolution of fungicide resistance and sustainable plant disease chemical management based on the fungicide dose chosen beyond that of potato blight, warranting further study on the resistance target gene.

## 1. Introduction

Plant diseases are one of the major threats to agricultural production worldwide [1], resulting in severe economic and crop yield loss yearly [2] and leading to health problems such as hunger and malnutrition, especially in the current era of continuing population growth [3] and escalating climate change [4]. Fungicides are the most convenient and effective approach for the comprehensive management of plant diseases in both smallholder and industrial productions of crops. As a result, the global use of fungicides (and bactericides) has continuingly increased over years, for example, from 393 million kilograms in 1990 to 530 million kilograms in 2018 (Food and Agriculture Organization of the United Nations; http://www.fao.org/).

However, the widespread and continued application of fungicides can exert significant selective pressure on pathogens, leading to the development of resistant phenotypes that can evade the inhibition of effective compounds in the fungicides, thus reducing their efficacy [5]. It is noteworthy that when resistant pathogens emerge, farmers often increase the dosage and frequency of fungicide application to compensate for the erosion of control effectiveness, which not only results in additional purchase and operating costs but also damages ecosystems and pollutes environments [6,7]. For example, increased chemical residues were found in aquatic ecosystems and on water surfaces of agricultural catchments throughout the growing season when fungicides were used more frequently and in greater amounts, which can rapidly accumulate and eventually become harmful to many nontarget organisms [8].

Many factors influence the development of pathogen resistance to fungicides, with chemical properties being one of the most important and of greatest concern. According to the mode of action, they can be classified into single-site fungicides and multi-site fungicides. Single-site fungicides target specific enzymes in critical biological pathways [9]. Thus, altering the structure or expression level of enzymes through the mutation of residues involved in the binding site as well as rearrangement and/or mutations in their promoter regions can greatly affect the efficacy of fungicides [10,11,12]. This type of resistance can easily emerge in pathogen populations [13] and has led to the complete withdrawals of many important fungicides from agricultural systems [14], such as multiple origins of resistance to metalaxyl used to control oomycete pathogens [15]. Unlike single-site fungicides, multi-site fungicides target numerous enzymes critical to biochemical, cellular, and ecological processes of pathogens [16], and the development of resistance to such fungicides involves many simultaneous changes in genome structure and pathogen expression, making them effective and durable against a wide range of pathogens, particularly when used in rotation and mixture with single-site fungicides [17].

In addition, genetic variation in pathogen populations is also thought to significantly affect the development of fungicide resistance [18]. Pathogens with higher population diversity may increase the opportunity of generating resistance mutations from their more diverse genetic backgrounds. Competition is one of the major forces driving species adaptation [19]. It can promote, inhibit, or sustain evolutionary processes, depending on whether the interactions among the players are synergistic, antagonistic, or neutral [20,21]. In nature, a single host plant is often simultaneously invaded by multiple pathogen strains of the same species. Due to advances in molecular technology in pathogen detection, this phenomenon has been increasingly confirmed in various plant–pathogen systems, such as *Alternaria alternata*, *Aspergillus flavus*, *Cryphonectria parasitica*, and *Sclerotinia sclerotiorum* on pear, cotton, Chinese chestnut, and oilseed rape, respectively [22,23,24,25]. High genetic variation in pathogen populations increases the opportunity for strains with different genetic backgrounds to co-infect a host plant, enhancing intraspecific competition of pathogens and changing their evolutionary resistance to fungicides.

A better understanding of the processes and mechanisms by which pathogens adapt to fungicides is critical for disease management and socio-ecological sustainability. However, research in this area has focused on predicting and preventing fungicide resistance in pathogen populations through retrospective approaches based on biochemical properties (e.g., the mode of action) and application strategies (e.g., fungicide rotation and mixture) of fungicides. It is rare to directly observe the development of fungicide resistance in pathogen populations and investigate how pathogen biology such as genetic variation influences the evolution. It is also rare to study the retention of acquired fungicide resistance after the removal of selective pressure. In this study, we address these concerns through an experimental evolution approach using the potato–*Phytophthora infestans* system.

Potato (*Solanum tuberosum*) is the third most consumed crop globally and makes a significant contribution to food security and social development [26]. Potato late blight caused by *P. infestans* is the most devastating disease of the crop, leading to the Irish famine in the 1840s and billions of US dollars in economic losses annually [27,28,29]. Although *R*-gene-mediated host resistance is the most economical and environmentally friendly way to control late blight, it can be quickly overcome due to the rapid evolution of the pathogen. Therefore, fungicides remain one of the most commonly used strategies to manage the disease, and the crop in many countries relies on more than 15 kinds of fungicide sprays in growing season to ensure good production [30,31,32,33].

Mancozeb is a typical multi-site protective fungicide [34] often used to prevent crop diseases including potato late blight. When exposed to water, the fungicide is decomposed to release ethylene bisisothiocyanate sulfide (EBIS), which is then converted to ethylene bisisothiocyanate (EBI) under UV light. Both EBIS and EBI are active toxins that interfere with enzymes containing sulfhydryl groups [34], thereby inhibiting spore germination of pathogens [35,36,37]. Although most agricultural chemists believe that pathogens are unlikely to develop mancozeb resistance, gradual enhancement in resistance to the fungicide has been documented in many species like *Colletotrichum aenigma* and *Fusarium avenaceum* [38,39].

To achieve our goal, we selected 10 different genotypes of *P. infestans* from a field and combined them in different ways to create 98 populations with a genotype complexity ranging from 1 to 10 (Figure S1, Table 1). The populations were acclimated to different concentrations of mancozeb for 200 consecutive days, and changes in mancozeb resistance measured by colony size were recorded according to the acclimation time and genotype complexity of the pathogen. The fitness cost of the acquired mancozeb resistance in the acclimated pathogens was assessed by association analysis of the resistance among different fungicide concentrations and with aggressiveness, respectively. The sustainability of the acquired resistance was evaluated in a reversal experiment in which the acclimated and unacclimated pathogens were placed on mancozeb-free plates for an additional 200 consecutive days. Genes and pathways associated with mancozeb resistance were identified by comparing the transcriptomes of resistant and sensitive isolates in the presence and absence of mancozeb.

The specific objectives of our study are to (1) explore how *P. infestans* adapts to mancozeb; (2) determine the influence of genotypes complexity in *P. infestans* population on the development of mancozeb resistance; (3) evaluate whether there is a fitness cost associated with *P. infestans* resistance to mancozeb after acclimation; (4) examine the specificity and sustainability of acquired resistance to mancozeb in *P. infestans*; and (5) excavate the genes and pathways associated with mancozeb resistance.

## 2. Materials and Methods

### 2.1. Phytophthora infestans Collection

A total of 653 *P. infestans* isolates collected from a field in Qujing, Yunnan, during the 2019 growing season were molecularly assayed using eight pairs of SSR primers (Pi02, Pi4B, Pi16, Pi33, Pi56, Pi63, Pi70, Pi89) [40,41]. SSR fragments in each locus were assigned to alleles based on their size, and alleles in the eight loci were combined in the same order to generate a multilocus profile for each isolate [42]. Isolates with the same multilocus profile were considered clones of the same genotype, and 10 self-fertility isolates with different multilocus profiles and biological characteristics (Figure S2) were selected for fungicide resistance analysis in the experiment. Detailed protocols for pathogen isolation, SSR amplification, and genotype assignment were described in our previous publications [43].

### 2.2. Generation of Initial P. infestans Populations

The 10 isolates retrieved from a long-term storage were cultured on rye B agar plates at 19 °C in the dark. After 10 days, each plate was flooded with 5 mL sterile water and scraped with an inoculation loop to release sporangia. The resulting suspension was filtered through a 50 µm nylon mesh and then collected into a 50 mL centrifuge tube. The sporangia concentration in each isolate was adjusted to an OD600 of 3 using a spectrophotometer, and 98 populations were formed with genotype complexity ranging from 1 to 10 (Figure S1, Table 1). Information on the isolate codes corresponding to all populations is detailed in Table S1. For the populations consisting of multiple genotypes, equal proportions of the sporangial suspension from each component were mixed.

### 2.3. Mancozeb Acclimation and Inoculation

In total, 30 µL of sporangia suspension was taken from each stock of the 98 populations, inoculated on a fresh rye B agar plate and cultured at 19 °C in the dark for 10 days to form a colony. A mycelia plug (8 mm in diameter) was taken from the margin of the colony and inoculated onto a new rye B agar plate either with 10 µg/mL (medium concentration of mancozeb, MMA) and 20 µg/mL (high concentration of mancozeb, HMA) of mancozeb (DRE-C14740000, CAS: 8018-01-7; dissolved in DMSO) or without mancozeb (CK) in a 9 cm Petri dish and kept in an incubator at 19 °C under dark conditions, with three replications for each treatment. Colonies were photographed on the 7th day after the inoculation with a camera (CANON-DS126832), and their sizes were subsequently digitized with ImageJ 1.5 [44]. On the 10th day after the inoculation, the pathogen was transferred to a fresh rye B agar plate with or without the supplementation of mancozeb (depending on its original treatment) by taking another 8 mm mycelia plug from the margin of the resulting colony. The acclimation process lasted for 200 days (Figure S1), leading to a total of 20 transfers for the experiment. Notably, the genotype complexity of the acclimated populations was not actively controlled during these transfers.

At the end of the 200-day experiment, the resulting pathogens were inoculated back onto rye B agar plates with the same fungicide concentrations (i.e., 0 µg/mL, 10 µg/mL, and 20 µg/mL) used for the acclimation process (Figure S1). These concentrations were carefully selected based on preliminary concentration–response assays [45], given that lower doses (<10 µg/mL) failed to produce measurable growth inhibition, while higher concentrations (>20 µg/mL) caused complete pathogen mortality, which would preclude resistance evolution studies. The colony sizes of the evolved pathogens were also measured on the 7th day post-inoculation, and the dose-dependent tradeoff of resistant adaptation was determined by observing the behavior of the pathogen in its acclimated and novel environments (fungicide concentration).

### 2.4. Testing the Persistence of Fungicide Resistance in the MMA-Acclimated Populations

The populations (Table 1) acclimated under 10 µg/mL mancozeb for 200 days were transferred to mancozeb-free rye B agar plates for an additional 200 days (Figure S1). On the 0th, 40th, 120th, and 200th days of the reversal experiment, the populations were re-exposed to the rye B agar plates supplemented with 10 µg/mL mancozeb and their colony sizes were measured as described in the earlier section. To avoid potential competition effects on fungicide resistance measurements, only single-genotype populations were used in the reversal experiment. Furthermore, the sustainability of the mancozeb resistance in the 10 single-genotype populations of the HMA population was not tested due to high mortality during acclimation. After acclimating 10 single-genotype isolates on mancozeb-free media for 400 days (Figure S1), the tolerance to 10 µg/mL mancozeb was measured at the 0th, 80th, 160th, 200th, 240th, 320th, and 400th days by transferring the 10 isolates to rye B agar plates supplemented with 10 µg/mL mancozeb and calculating the colony size to serve as a control group.

### 2.5. Aggressiveness Determination of MMA, HMA, and CK Treatments

The aggressiveness of *P. infestans* was quantified by the lesion size induced on detached leaflets as described previously [46]. Bintje, a variety with no known *R*-genes, was used for the test. Briefly, after 200 days of acclimation, the mycelia plugs (8 mm in diameter) taken from the MMA, HMA, and CK populations were inoculated on the abaxial side of fully expanded leaflets of Bintje grown in a greenhouse. The inoculated leaflets were placed on 1% water agar plates and kept at 19 °C in an incubator supplemented with 16 h of light daily. Lesions were photographed with a camera (CANON-DS126832) at the 6th day after inoculation, and their sizes were quantified using ImageJ 1.5 [44]. Each *P. infestans* population was replicated three times. The entire measurement of aggressiveness was carried out by the same person to reduce artificial errors.

### 2.6. Preparation for Transcriptome Sequencing

After 200 days of mancozeb acclimation, one isolate was selected from the CK and MMA treatments, respectively, each with a stable sensitive and resistant phenotype. For convenience in subsequent descriptions, the CK-derived isolate showing less resistance to mancozeb was designated as the S (sensitive) isolate, while the MMA-derived isolate that exhibited high resistance to mancozeb was designated as the R (resistant) isolate The S isolate was inoculated onto rye B agar plates supplemented with no mancozeb and with 10 µg/mL mancozeb with 8 mm mycelia plugs, labeled as S0 and S10, respectively. Similarly, the R isolate was also inoculated onto rye B agar plates containing no mancozeb and 10 µg/mL mancozeb through 8 mm mycelia plugs and was marked as R0 and R10, respectively. The experiment was replicated three times, leading to 12 plates for the four treatments. On the 6th day of cultivation at 19 °C in the dark, mycelia from each plate were scraped from the media using a sterile glass slide, transferred into a sterilized 2 mL centrifuge tube, and stored in −80 °C refrigerator after quick freezing with liquid nitrogen. The 12 specimens (three replicates for each of four treatments) were sent to Biomarker Technologies Company for transcriptome sequencing.

### 2.7. Phenotypic Data Analysis

Mancozeb resistance in the pathogen was inferred from colony size. Analysis of variance (ANOVA) was performed using the general linear model procedure in IBM SPSS Statistics (Version 27.0) by partitioning variance components attributable to population, fungicide concentration, acclimation time, and their interaction. The least significant difference (LSD) was used to compare colony and lesion area among different treatments. The effects of acclimation time and genotype complexity on the development of mancozeb resistance were evaluated using Pearson and Spearman correlation embedded in Origin 2021, respectively.

### 2.8. Transcriptome Data Analysis

Pearson correlation coefficients were employed to evaluate the association between the transcriptome data (FPKM) of four treatments (S0, S10, R10, R0) using the prcomp and cor functions of R software (Version 3.6.4), respectively. Differential expressions between treatments were analyzed using the DESeq2, which provides a statistical determination based on the negative binomial distribution. The resulting *p* values were adjusted using the method of Benjamini and Hochberg to control for false discovery rate, and only genes discovered by DESeq2 with an adjusted *p*-value < 0.01 and a fold change ≥2 were assigned as differentially expressed genes (DEGs). KEGG [47] is a database resource for understanding the high-level functions and utility of biological systems. Here, we used the KOBAS [48] database and clusterProfiler software (Version 3.18.1) to test the statistical enrichment of DEGs in KEGG pathways. To discover expression trends of DEGs, co-expression was analyzed using *k*-means clustering (Figure S3). All analyses of the transcriptome data were completed on BMKCloud (www.biocloud.net).

## 3. Results

### 3.1. Gradual Increase in Mancozeb Resistance over Acclimation Time in P. infestans

**Figure 1:**
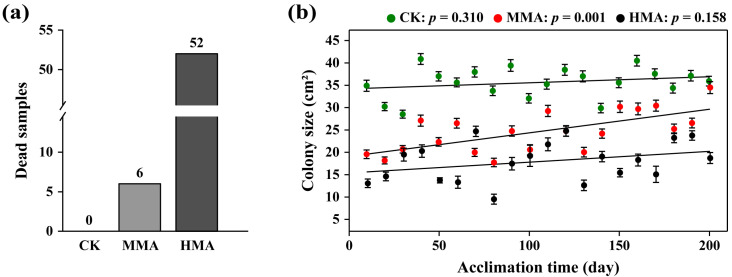
Mancozeb resistance of *Phytophthora infestans* in response to long time acclimation: (**a**) The average number of dead samples in the CK (control), 10 µg/mL (MMA), and 20 µg/mL (HMA) mancozeb treatments after 200 days of acclimation. (**b**) Change in mancozeb resistance measured by colony size over acclimation time in the CK, MMA, and HMA treatments. Association between mancozeb resistance and acclimation time was evaluated by Pearson correlation. In panel (**b**), each data point is the average value of the colony size of 98 populations with a 95% confidence interval. Dead samples were removed from analysis.

Colony size was quantified for all 98 populations (n = 294 measurements, three replicates per population) on the 7th day after each transfer (10 days/transfer) over the acclimation of 200 days. Of the 98 populations, 0, 6, and 52 died after 200 days of acclimation on rye B agar plates supplemented with 0 µg/mL (CK), 10 µg/mL (MMA), or 20 µg/mL (HMA), respectively (Figure 1a). Univariate analysis of variance indicated significant contributions of isolate, fungicide concentration, acclimation time, isolate × fungicide concentration, isolate × acclimation time, fungicide concentration × acclimation time, and isolate × fungicide concentration × acclimation time to the resistance of *P. infestans* to mancozeb measured by colony size (Table S2).

The average colony size of the CK populations remained stable at around 35.0 cm^2^ throughout the 200-day acclimation time period (Pearson: *r* = 0.239, *p* = 0.310; Figure 1b), and this stability in growth patterns was observed consistently across all ten genotype complexity levels (Table S3), demonstrating consistent growth characteristics in the absence of mancozeb selection. Initially, the average colony size of the MMA populations was approximately 20 cm^2^, which was 15.3 cm^2^ (43%) smaller than that of the CK populations. However, after 200 days of acclimation, the average colony size of the MMA populations reached 35.5 cm^2^, which was almost equivalent (97%) to that of CK populations (Figure 1b). Moreover, the average colony size of the MMA populations gradually increased over time as indicated by the positive and significant correlation between the colony size and the acclimation time (Pearson: *r* = 0.665, *p* = 0.001), and the same pattern was also observed across all 10 genotype complexity levels (Table S3), suggesting an increase in mancozeb resistance in *P. infestans* after prolonged exposure to medium concentration (10 µg/mL) of the fungicide (Figure 1b).

The initial average colony size of the HMA populations was 13 cm^2^, 22.3 cm^2^ (63%) and 7 cm^2^ (35%) smaller than that of the CK and MMA populations, respectively. After 200 days of acclimation, the colony size of the HMA populations reached 18.7 cm^2^, 49% and 47% smaller than that of CK and MMA populations, respectively. However, the average colony size in these populations only marginally increased with acclimation time (Pearson: *r* = 0.328, *p* = 0.158; Figure 1b).

### 3.2. Genotype Complexity Benefits the Resistance of P. infestans to Mancozeb

**Figure 2:**
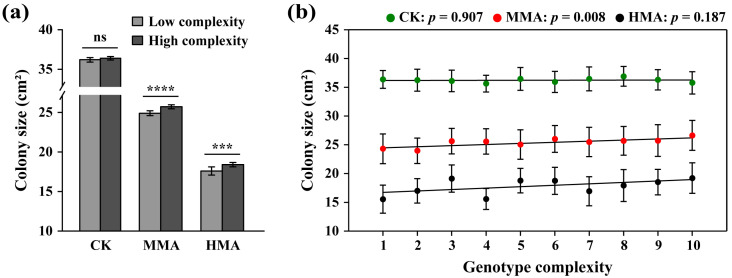
Contribution of genetic diversity in *Phytophthora infestans* to the development of mancozeb resistance as measured by colony size after 200 days of acclimation: (**a**) Least significant difference (LSD) in mancozeb resistance between treatments with high (6–10-genotype mixture) and low (1–5-genotype mixture) genotype complexity. ****, ***, and ns above error bars (standard error) indicate *p* < 0.0001, *p* < 0.001, and *p* > 0.05, respectively. (**b**) Association (Spearman correlation) between mancozeb resistance in the CK, MMA, and HMA treatments and genotype complexity. In panel (**b**), each data point indicates the average value of the colony size of 20 transfers with a 95% confidence interval. Dead samples were removed from analysis.

Pathogens with higher genetic variation usually adapt better to ecological stress such as host resistance and fungicides. Here, we tested the hypothesis by comparing the colony size of the *P. infestans* populations with different genotype complexities after 200 days of continuous culture on the media supplemented with mancozeb fungicide and without the fungicide. We first grouped the 98 populations into low (1–5-genotype mixture) and high (6–10-genotype mixture) genotype complexity (Table 1) and found that mancozeb resistance in the pathogen measured by colony size was similar between the CKs with high genotype complexity and low genotype complexity (Figure 2a). However, under continuous mancozeb stress, resistance to the fungicide was significantly higher in the MMA and HMA populations with high genotype complexity than that with low genotype complexity (Figure 2a). Further analysis by Spearman correlation showed that mancozeb resistance was not associated with genotype complexity in the CK (*r* = 0.042, *p* = 0.907; Figure 2b) but positively associated with genotype complexity in the MMA populations (*r* = 0.782, *p* = 0.008; Figure 2b). Although a positive correlation was also found in the HMA populations, it was not significant statistically (*r* = 0.455, *p* = 0.187, Figure 2b).

### 3.3. Tradeoff Analyses in Mancozeb Resistance of P. infestans

**Figure 3:**
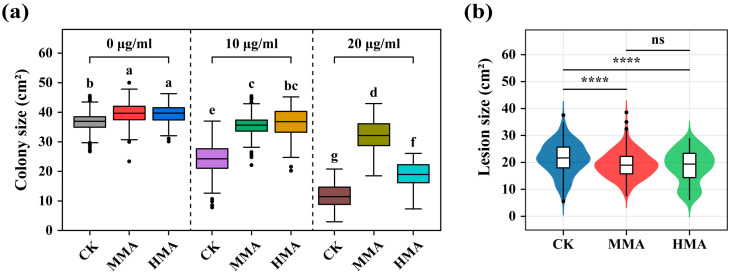
The tradeoff associated with *Phytophthora infestans* resistance to mancozeb after 200 days of acclimation on CK, MMA, and HMA media: (**a**) Differences within and between concentrations in mancozeb resistance measured by colony size. Boxes marked with different letters indicate significant differences at *p* < 0.05 according to the least significant difference (LSD) test. (**b**) Comparison of aggressiveness measured by lesion size between CK, MMA, and HMA treatments. ****, *p* < 0.0001; ns, *p* > 0.05. In panels (**a**,**b**), each data point indicates 98 populations. Dead samples were removed from analysis. Box plots: center line, median; box limits, upper and lower quartiles; whiskers, 1.5× interquartile range.

Dose-dependent tradeoff of mancozeb resistance in *P. infestans* was assessed by transferring the populations acclimated for 200 days to the three-fungicide concentrations used for the acclimation experiment. Mancozeb resistance was higher in the MMA and HMA populations than in the CK populations at all three fungicide concentrations (Figure 3a). At the high concentration (20 µg/mL), MMA populations had a higher mancozeb resistance than the HMA populations. But at the low concentration (10 µg/mL), there was no difference in mancozeb resistance between the two types of populations. Again, this pattern was consistent across the 10 genotype complexity levels (Table S4). Although the fungicide resistance of the acclimated populations generally reduced with increasing mancozeb concentrations in the cross-inoculation experiment, the extent of the reduction varied greatly among the CK, MMA, and HMA populations (Figure 3a). The CK populations showed the greatest reduction in resistance, followed by HMA populations, while MMA populations showed the least reduction. Furthermore, the acclimated populations with higher mancozeb resistance at one mancozeb concentration always showed higher resistance at another mancozeb concentration, suggesting the dose-independent resistance of *P. infestans* to the fungicide.

Aggressiveness measured by lesion size was also determined for the CK, MMA, and HMA populations. It was found that lesion size was significantly smaller in the MMA (19.1 cm^2^) and HMA (18.5 cm^2^) populations than in the CK (21.4 cm^2^) populations (*p* < 0.0001, Figure 3b) but not different between the MMA and HMA populations (Figure 3b). This result suggests that the development of mancozeb resistance incurs a fitness cost to aggressiveness of *P. infestans* that is independent of the mancozeb concentration.

### 3.4. Acquisition of Mancozeb Resistance Through Acclimation Is Reversible After Relaxing Selective Pressure

**Figure 4:**
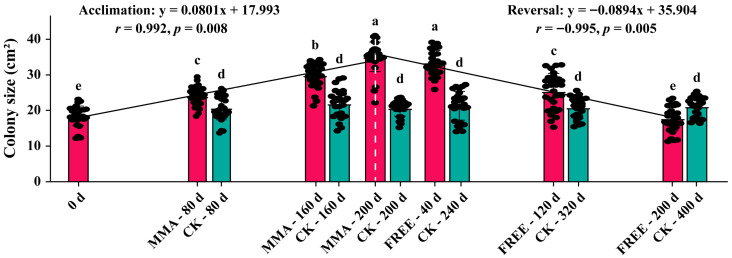
Changes in mancozeb resistance in the 10 single-genotype *Phytophthora infestans* populations acclimated on 0 µg/mL mancozeb (CK), 10 µg/mL mancozeb (MMA, left), and after the return of the MMA-acclimated populations to mancozeb-free rye B agar plates (right). Error bars (standard error) marked with different letters indicate significant difference at *p* < 0.05 according to the least significant difference (LSD) test. The associations of the loss and acquirement of resistance with acclimation time were assessed by Pearson correlation. Each data point includes 10 single-genotype populations with 3 replications.

To check whether the acquisition of mancozeb resistance after 200 days of acclimation on 10 µg/mL mancozeb was reversible or not, the 10 populations, each with a single genotype (Table 1) acclimated on 10 µg/mL mancozeb, were transferred to mancozeb-free rye B agar plates for another 200 days, and resistance to 10 µg/mL mancozeb was measured on the 40th, 120th, and 200th days of the reversal experiment. The result shows that resistance to mancozeb in the acclimated populations continued to decline over time (Figure 4). At the 40th, 120th, and 200th days after being transferred to mancozeb-free rye B agar plates (Figure 4, right part), the average colony size in the 10 acclimated populations decreased to 33.4, 25.4, and 17.7 cm^2^, almost the same as their colony sizes on the 40th (MMA-160d), 120th (MMA-80d), and 200th (MMA-0d) day prior the end of the acclimation experiment (Figure 4, left part). Meanwhile, the rate of resistance gain during the acclimation (slope = 0.0801) and the rate of resistance loss during the reversal experiment (slope = −0.0894) were almost identical. Therefore, we believe the acquired resistance to mancozeb in *P. infestans* is reversible when the fungicide is absent for a period of time.

### 3.5. ATP-Binding Cassette Transporters (ABC Transporters) and Endocytosis Pathways Contribute Greatly to the Development of Mancozeb Resistance in P. infestans

**Figure 5:**
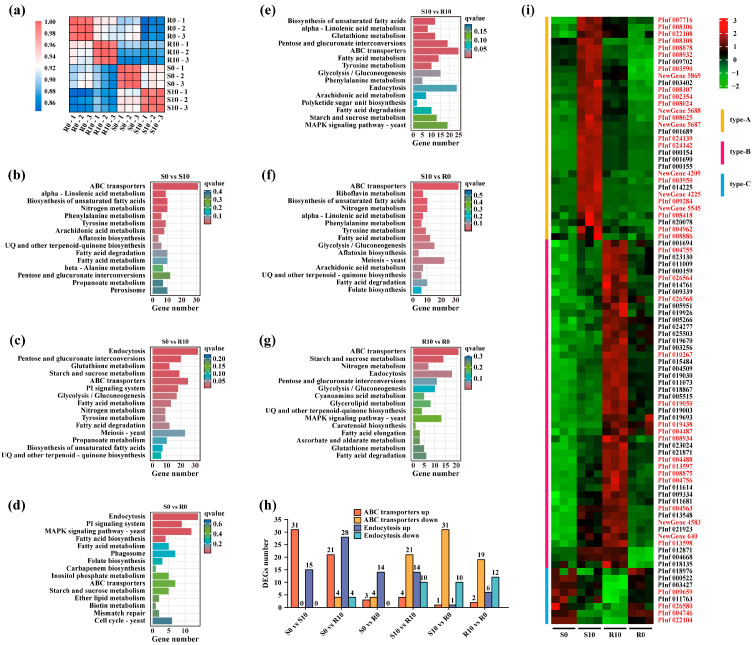
Transcriptome patterns of *Phytophthora infestans* after 200 days of acclimation on media with and without mancozeb supplementation: (**a**) Pearson correlation coefficients (PCCs) between four treatments (S0, S10, R0, and R10), each with three replicates. (**b**–**g**) KEGG enrichment analysis of pairwise comparison between four treatments. Only the top 15 pathways in each comparison are shown. (**h**) The number of differentially expressed genes (DEGs, including up- and downregulation) associated with ABC transporters and endocytosis pathways enriched from the six pairwise comparisons between treatments. (**i**) Heat map of the 87 DEGs associated with ABC transporters (44) and endocytosis (43) pathways at four distinct treatment conditions. Red and black gene IDs indicate DEGs associated with ABC transporters and endocytosis pathways, respectively. S: isolate acclimated in CK treatment for 200 days, highly sensitive to mancozeb; R: isolate acclimated in MMA treatment for 200 days, highly resistant to mancozeb; S0: S isolate inoculated onto rye B agar plates with no mancozeb; S10: S isolate inoculated onto rye B agar plates with 10 µg/mL mancozeb; R0: R isolate inoculated onto rye B agar plates with no mancozeb; R10: R isolate inoculated onto rye B agar plates with 10 µg/mL mancozeb.

To identify genes and pathways contributing to the development of *P. infestans* resistance to mancozeb, we cross-inoculated the evolved S and R isolates on rye B agar plates supplemented with 0 and 10 µg/mL mancozeb, generating a total of four treatments (i.e., S0, S10, R0, and R10) and analyzed their transcriptome patterns. Correlation analysis showed the transcriptome profiles of three replicates in each treatment were highly reproducible (Figure 5a), indicating that the overall transcriptome data are reliable for further analysis to discover genes and pathways associated with the development of mancozeb resistance in *P. infestans*.

Using KEGG enrichment analysis for the six combinations (S0 vs. S10, S0 vs. R10, S0 vs. R0, S10 vs. R10, S10 vs. R0, and R10 vs. R0), it was found that the ABC transporters and endocytosis pathways were significantly enriched in almost all of these combinations (Figure 5b–g), and both up- and downregulated genes associated with the two pathways were enriched in four combinations (S0 vs. R10, S10 vs. R10, S10 vs. R0, and R10 vs. R0; Figure 5h). When gene co-expression trend analysis was performed on all DEGs in the six combinations (Figure S3), 44 ABC transporters-associated genes and 43 endocytosis-associated genes were identified.

A heat map created using the expression levels of the 87 genes in four treatments (S0, S10, R10 and R0; Figure 5i) showed that these 87 genes were divided into three categories: (A) Their expression is significantly upregulated when *P. infestans* is first exposed to mancozeb but begins to be downregulated as the acclimation time increases, and continues to be downregulated when *P. infestans* returns to being mancozeb-free. (B) Their expression is upregulated when *P. infestans* is first exposed to mancozeb, and continues to be upregulated as the acclimation time increases, but is downregulated when *P. infestans* returns to being mancozeb-free. And (C) their expression is downregulated when *P. infestans* is first exposed to mancozeb, continues to be downregulated as the acclimation time increases, and is upregulated when *P. infestans* returns to being mancozeb-free. A heat map analysis of the DEGs also revealed distinct expression patterns associated with mancozeb response. DEGs in ABC transporters and endocytosis pathways showing differential expression in both S0 vs. S10 and R10 vs. R0 comparisons likely represent transient stress responses to mancozeb exposure. In contrast, DEGs consistently observed in both S0 vs. R0 and S10 vs. R10 comparisons were strongly associated with the resistant phenotype. Based on these patterns, we identified 14 high-confidence candidate genes (5 ABC transporter-related and 9 endocytosis-related genes, Table S5) as key molecular determinants of mancozeb resistance in *P. infestans*. These candidate genes demonstrated stable expression differences between sensitive and resistant isolates regardless of immediate fungicide exposure, suggesting they may underlie constitutive resistance mechanisms. These results suggest that the DEGs associated with ABC transporters and endocytosis pathways regulate the development of mancozeb resistance in *P. infestans* through different expression patterns.

## 4. Discussion

The emergence of fungicide resistance in pathogens is a serious worldwide problem [49], with deep implications for food security as population growth requires more food to feed society [26], and climate change may exacerbate disease epidemics [4] and complicate management strategies [50]. The evolution of fungicide resistance can arise from mutations and/or altered expression of target genes. In this study, an experimental evolution approach was used to acclimate a series of genetically diverse *P. infestans* populations to continuous selection pressure with mancozeb for 200 days. The result shows that the colony size of *P. infestans* gradually increases over the acclimation time (Figure 1b), indicating that the pathogen can develop resistance to mancozeb, which is contrary to the theoretical views of many agricultural chemists on this non-specific fungicide [17,34], but consistent with the field observation [51]. Interestingly, we found that resistance to high mancozeb stress was not as robust as to medium mancozeb stress, as indicated by the overall smaller colony size from HMA pathogens than from MMA pathogens, especially when they were re-inoculated to 20 µg/mL mancozeb (Figure 3a). This result suggests that high doses inhibit pathogen evolution to fungicide resistance, either because small populations associated with high mortality (Figure 1a) reduce the generation of beneficial mutations for resistance, or that resistance to high doses imposes severe fitness penalties that mask the benefits of the resistance. This emphasizes the importance of using adequate fungicide doses in practice to achieve good disease control and reduce the development of fungicide resistance.

The resistance of *P. infestans* to mancozeb increased with increasing genotype complexity in the populations (Figure 2), consistent with the theory hypothesizing that genetic variation benefits the adaptation of species to ecological stress [52]. Genetic variation in populations arises primarily through sexual recombination, which is believed to be triggered by environmental stresses for pathogenic fungi with a facultative sexual stage [53], and somatic mutations [54] that are strongly linked to population size. Climate change is an unprecedented and ongoing event that may lead to an increase in average temperatures by 4 °C compared to preindustrial times (1850–1919) at the end of the 21st century (IPCC, 2014), accompanied by more frequent extreme weather events [55]. Rising temperatures may promote the growth of species including plant pathogens, thereby expanding their population sizes, while stress induced by extreme weather events may stimulate the sexual reproduction of fungal pathogens [56]. In *P. infestans*, we found that field populations increasingly self-fertilize [57], which may facilitate the sexual reproduction of the pathogen to generate more genetic variation for adaptation. Therefore, we hypothesize that the evolution of fungicide resistance in ecosystems may accelerate. Future research should explore more with these climate change issues, both theoretically and empirically, for food security and socio-ecological sustainability.

Apparently, resistance to mancozeb in *P. infestans* is dose-independent (Figure 3a). This observation reflects well the expected resistance mechanisms of the fungicide. Mancozeb is a non-specific (multi-site) fungicide. The resistance is largely triggered by the increased efflux by the membrane transporters of pathogens [9]. Pathogens that work well at excreting and repelling the fungicide in one dosage environment would do so equally well in another dosage environment.

The sustainability of acquired fungicide resistance in pathogens has received widespread academic, practical, and public attention due to its economic and ecological importance. Here, we unambiguously demonstrate, for the first time, that resistance to mancozeb in the potato blight pathogen is reversible. During the 400-day experiment, the resistance of *P. infestans* to mancozeb gradually built up within the first 200 days of continuous treatment with the fungicide but eroded within the second 200 days after the complete removal of the fungicide (Figure 4). Furthermore, the symmetrical distribution of resistance gains and losses before and after fungicide removal indicates that these two events occur at nearly identical rates. This reversibility may be attributed to the fitness costs associated with the development of mancozeb resistance. The evolution of fungicide resistance involves complex tradeoffs that can significantly impact pathogen fitness through multiple mechanisms. Our study specifically examined two key types: (1) concentration-dependent tradeoffs across fungicide doses and (2) functional tradeoffs between resistance and virulence traits. While we did not observe a clear dose-dependent tradeoff and did not evaluate the aggressiveness difference between 200 days after acclimation and 200 days after the reversal experiment, our results demonstrate that mancozeb resistance in *P. infestans* incurs significant fitness costs, particularly in ecological functions like aggressiveness (Figure 3b). Therefore, when selection from mancozeb is released, *P. infestans* may gradually restore its aggressiveness at the expense of reducing mancozeb resistance, a phenomenon widely documented in host–pathogen interactions [58,59]. Taken together, this result highlights the possibility of recycling fungicides in practice. However, the exact timing of the discontinuation for a particular fungicide depends on the biology of its associated pathogens and its chemistry.

Efflux transporters are often silent or weakly expressed, but their expression can rapidly increase when exposed to drugs [60]. Transcriptome analysis confirms the involvement of transporters in the development of mancozeb resistance in *P. infestans* (Figure 5). Among them, the ABC superfamily is one of the major transporters located on the cell membrane. Low substrate specificity enables them to transport a variety of structurally diverse compounds [61]. It has been well documented that ABC transporters are mediators of both single- and multi-drug resistance in plant pathogens [62,63,64], and oomycetes like *P. infestans* possess an expanded repertoire of ABC transporters compared to ascomycetes and basidiomycetes [65]. While multi-site fungicide resistance mechanisms remain poorly characterized, the ABC transporter gene *AG1IA_06082* has been shown to confer chlorothalonil resistance in *Rhizoctonia solani* [64], providing precedent for our findings. Indeed, during the acclimation process, the expression of some genes associated with ABC transporter pathways was continuously up- or downregulated (Figure 5i). Within the heat map range, the DEGs of ABC transporters exist in both S0 vs. R0 and S10 vs. R10, probably related to resistant phenotypes closer than those in S0 vs. S10 and R10 vs. R0 (transient expression). Consequently, we identified five DEGs of ABC transporters (Table S5) that are vital for the resistance of *P. infestans* to mancozeb, but the specific functions of these genes need to be further verified.

Transcriptome analysis suggests that endocytic proteins may also be involved in the development of mancozeb resistance in *P. infestans* (Figure 5). Endocytosis enables cells to absorb macromolecule nutrients from the surrounding medium [66], which is required for hyphal growth in filamentous fungi [67]. Many of these genes were differentially expressed between the different treatments of mancozeb (Figure 5b–g). Mancozeb inhibits the hyphal growth of *P. infestans*. Upregulating the expression of genes associated with endocytic processes may facilitate the polar growth of *P. infestans* hyphae, thereby offsetting the inhibitory effect from mancozeb. On the other hand, it is possible that *P. infestans* can alleviate the toxicity of mancozeb by enhancing the endocytosis and hydrolysis of certain macromolecular substances, as documented in parasite resistance to artemisinin [68]. Recent studies have demonstrated that endocytosis facilitates reactive oxygen species (ROS) scavenging in *Corynespora cassiicola* by promoting mitophagy during trifloxystrobin exposure [69]. Given that mancozeb similarly induces mitochondrial ROS production in fungi, we propose an analogous protective mechanism in *P. infestans*, where endocytosis may contribute to mancozeb resistance by enhancing ROS clearance. Our transcriptomic analysis identified nine endocytosis-related DEGs, which were included in both S0 vs. R0 and S10 vs. R10 comparisons (Table S5, heat map analysis). Like the ABC transporters discussed previously, these candidate genes exhibit expression profiles strongly associated with the resistant phenotype, suggesting they may function coordinately to mitigate fungicide stress. Future studies should validate the specific roles of these endocytic components in mancozeb detoxification and ROS management.

In conclusion, we found that the development of mancozeb resistance can be rapidly established in *P. infestans*. This establishment is related to ABC transporters and endocytic proteins and is reversible. The observed reversibility of resistance after the relaxation of selective pressure may explain the sustained durability of mancozeb in agricultural applications and indicates that *P. infestans* is unlikely to produce stable mancozeb-resistant mutants, at least under 200 days of acclimation. Our results provide novel insights into the evolution of fungicide resistance and plant disease management beyond potato blight and warrant further study.

## Figures and Tables

**Table 1:** Isolate (a–j) combination in 98 *Phytophthora infestans* populations with genotype complexity ranging from 1 to 10.

Genotype Complexity	Grouping	Number	Isolate Combination
**1**	Low	10	a, b, c, d, e, f, g, h, i, j
**2**		11	ab, bc, cd, de, ef, fg, gh, hi, ij, ja, dh
**3**		11	abc, bcd, cde, def, efg, fgh, ghi, hij, ija, jab, jdf
**4**		11	abcd, bcde, cdef, defg, efgh, fghi, ghij, hija, ijab, jabc, cegi
**5**		11	abcde, bcdef, cdefg, defgh, efghi, fghij, ghija, hijab, ijabc, jabcd, bdfhj
**6**	High	11	abcdef, bcdefg, cdefgh, defghi, efghij, fghija, ghijab, hijabc, ijabcd, jabcde, abefij
**7**		11	abcdefg, bcdefgh, cdefghi, defghij, efghija, fghijab, ghijabc, hijabcd, ijabcde, jabcdef, abceghj
**8**		11	abcdefgh, bcdefghi, cdefghij, defghija, efghijab, fghijabc, ghijabcd, hijabcde, ijabcdef, jabcdefg, bcdfghij
**9**		10	abcdefghi, bcdefghij, cdefghija, defghijab, efghijabc, fghijabcd, ghijabcde, hijabcdef, ijabcdefg, jabcdefgh
**10**		1	abcdefghij

Note: “a–j” indicate 10 single-genotype isolates; “ab” indicates the mixture of two isolates “a” and “b”, and so on; “abcdefghij” indicates the mixture of 10 isolates “a”, “b”, “c”, “d”, “e”, “f”, “g”, “h”, “i”, and “j”.

## Data Availability

The phenotype data needed for the evaluation of the conclusions in the paper were deposited in Dryad (http://datadryad.org/share/vUkODJ0GJqpT1sY-zC9TBHIK5jkgFU9jYWxmLIC6Jws), accessed on 28 August 2025. The transcriptome data were deposited with the China National Center for Bioinformation under accession number PRJCA035287 (https://ngdc.cncb.ac.cn/gsa/browse/CRA022373), accessed on 7 August 2025.

## Supplementary Materials

The following supporting information can be downloaded at https://www.mdpi.com/article/10.3390/jof11090643/s1: Figure S1: Experimental flow chart. Figure S2: Biological characteristics of the 10 original isolates. Figure S3: Co-expression trends from DEGs among the six combinations. A total of 4223 DEGs from six combinations (S0 vs. S10, S0 vs. R10, S0 vs. R0, S10 vs. R10, S10 vs. R0, and R10 vs. R0) were selected and classified into eight modules (a–h). Table S1: The isolate codes of 98 populations with genotype complexity ranging from 1 to 10. Table S2: Univariate analysis of variance in acclimation time, fungicide concentration, isolate, and interaction of the 98 populations of *Phytophthora infestans*. Table S3: Pearson correlation analysis between average colony size of the 10 *Phytophthora infestans* populations with different genotype complexities and acclimation times under CK, MMA, and HMA conditions. Table S4: Least significant difference (LSD) test for mancozeb (0, 10, 20 µg/mL) resistance of 10 *Phytophthora infestans* populations with different genotype complexities after 200 days of acclimation on CK, MMA, and HMA media. Note: Different letters in the same column indicate significant differences at *p* < 0.05. Table S5: The 14 candidate genes included in ABC transporters or endocytosis pathways.
