# Supplementary material for: Rise and Fall of Phytophthora infestans Resistance to Non-Specific Fungicide in Experimental Populations
Source: J Fungi (Basel). 2025 Aug 30;11(9):643. doi: 10.3390/jof11090643 (PMC12470341; doi:10.3390/jof11090643)
Supplement: Supplementary file 1 [file jof-11-00643-s001.zip › Supplementary Tables/Table S1.pdf]

Table S1 The isolate codes of 98 populations with genotype complexity ranging from 1 to 10.

Isolate code of genotype complexity 1	Population of genotype complexity 1	Isolate code of genotype complexity 2	Population of genotype complexity 2	Isolate code of genotype complexity 3	Population of genotype complexity 3	Isolate code of genotype complexity 4	Population of genotype complexity 4	Isolate code of genotype complexity 5	Population of genotype complexity 5	Isolate code of genotype complexity 6	Population of genotype complexity 6	Isolate code of genotype complexity 7	Population of genotype complexity 7	Isolate code of genotype complexity 8	Population of genotype complexity 8	Isolate code of genotype complexity 9	Population of genotype complexity 9	Isolate code of genotype complexity 10	Population of genotype complexity 10
1-1	a	2-1	ab	3-1	abc	4-1	abcd	5-1	abcde	6-1	abcdef	7-1	abcdefg	8-1	abcdefgh	9-1	abcdefghi	10-1	abcdefghij
1-2	b	2-2	bc	3-2	bcd	4-2	bcde	5-2	bcdef	6-2	bcdefg	7-2	bcdefgh	8-2	bcdefghi	9-2	bcdefghij		
1-3	c	2-3	cd	3-3	cde	4-3	cdef	5-3	cdefg	6-3	cdefgh	7-3	cdefghi	8-3	cdefghij	9-3	cdefghija		
1-4	d	2-4	de	3-4	def	4-4	defg	5-4	defgh	6-4	defghi	7-4	defghij	8-4	defghija	9-4	defghijab		
1-5	e	2-5	ef	3-5	efg	4-5	efgh	5-5	efghi	6-5	efghij	7-5	efghija	8-5	efghijab	9-5	efghijabc		
1-6	f	2-6	fg	3-6	fgh	4-6	fghi	5-6	fghij	6-6	fghija	7-6	fghijab	8-6	fghijabc	9-6	fghijabcd		
1-7	g	2-7	gh	3-7	ghi	4-7	ghij	5-7	ghija	6-7	ghijab	7-7	ghijabc	8-7	ghijabcd	9-7	ghijabcde		
1-8	h	2-8	hi	3-8	hij	4-8	hija	5-8	hijab	6-8	hijabc	7-8	hijabcd	8-8	hijabcde	9-8	hijabcdef		
1-9	i	2-9	ij	3-9	ija	4-9	ijab	5-9	ijabc	6-9	ijabcd	7-9	ijabcde	8-9	ijabcdef	9-9	ijabcdefg		
1-10	j	2-10	ja	3-10	jab	4-10	jabc	5-10	jabcd	6-10	jabcde	7-10	jabcdef	8-10	jabcdefg	9-10	jabcdefgh		
		2-11	dh	3-11	jdf	4-11	cegi	5-11	bdfhj	6-11	abefij	7-11	abceghj	8-11	bcdfghij				
