# Supplementary material for: Rise and Fall of Phytophthora infestans Resistance to Non-Specific Fungicide in Experimental Populations
Source: J Fungi (Basel). 2025 Aug 30;11(9):643. doi: 10.3390/jof11090643 (PMC12470341; doi:10.3390/jof11090643)
Supplement: Supplementary file 1 [file jof-11-00643-s001.zip › Supplementary Tables/Table S2.pdf]

Table S2 Univariate analysis of variance in acclimation time, fungicide concentration, isolate and interaction of 98 populations of *Phytophthora infestans*.

Source	DF	MS	F-statistic	*P*-value
**Acclimation time**	19	5701.682	2227.604	< 0.0001
**Fungicide concentration**	2	311038.084	121520.228	< 0.0001
**Isolate**	97	357.267	139.581	< 0.0001
**Acclimation time x Fungicide concentration**	38	2532.076	989.263	< 0.0001
**Acclimation time x Isolate**	1843	32.284	12.613	< 0.0001
**Fungicide concentration x Isolate**	142	149.403	58.371	< 0.0001
**Acclimation time x Fungicide concentration x Isolate**	2599	31.17	12.178	< 0.0001
