# Supplementary material for: Rise and Fall of Phytophthora infestans Resistance to Non-Specific Fungicide in Experimental Populations
Source: J Fungi (Basel). 2025 Aug 30;11(9):643. doi: 10.3390/jof11090643 (PMC12470341; doi:10.3390/jof11090643)
Supplement: Supplementary file 1 [file jof-11-00643-s001.zip › Supplementary Tables/Table S3.pdf]

**Table S3** Pearson correlation analysis between colony size of 10 *Phytophthora infestans* populations with different genotype complexities and acclimation time under CK, MMA and HMA conditions.

| **Genotype complexity** | **CK** | | **MMA** | | **HMA** | |
|---|---|---|---|---|---|---|
| | ***r*** | ***p*** | ***r*** | ***p*** | ***r*** | ***p*** |
| **1** | 0.441 | 0.052 | 0.616 | 0.004 | 0.238 | 0.313 |
| **2** | 0.252 | 0.283 | 0.638 | 0.002 | 0.256 | 0.275 |
| **3** | 0.416 | 0.068 | 0.633 | 0.003 | 0.443 | 0.051 |
| **4** | 0.134 | 0.573 | 0.693 | < 0.001 | 0.334 | 0.151 |
| **5** | 0.210 | 0.374 | 0.713 | < 0.001 | 0.319 | 0.170 |
| **6** | 0.208 | 0.378 | 0.696 | < 0.001 | 0.482 | 0.031 |
| **7** | 0.115 | 0.630 | 0.534 | 0.015 | 0.353 | 0.127 |
| **8** | 0.123 | 0.607 | 0.539 | 0.014 | 0.083 | 0.729 |
| **9** | 0.101 | 0.673 | 0.598 | 0.005 | 0.232 | 0.326 |
| **10** | 0.305 | 0.192 | 0.532 | 0.016 | 0.059 | 0.805 |
