# Supplementary material for: Rise and Fall of Phytophthora infestans Resistance to Non-Specific Fungicide in Experimental Populations
Source: J Fungi (Basel). 2025 Aug 30;11(9):643. doi: 10.3390/jof11090643 (PMC12470341; doi:10.3390/jof11090643)
Supplement: Supplementary file 1 [file jof-11-00643-s001.zip › Supplementary Tables/Table S4.pdf]

Table S4 Least significant difference (LSD) test for mancozeb (0, 10, 20 μg/ml) resistance of 10 *Phytophthora infestans* populations with different genotype complexity after 200 days of acclimation on CK, MMA and HMA media. Note: different letters in the same row indicate significant differences at $p < 0.05$.

Genotype complexity	0 μg/ml (colony size - cm^2)			10 μg/ml (colony size - cm^2)			20 μg/ml (colony size - cm^2)		
	**CK**	**MMA**	**HMA**	**CK**	**MMA**	**HMA**	**CK**	**MMA**	**HMA**
**1**	34.936^{b}	40.836^{a}	40.013^{a}	23.945^{d}	34.995^{b}	34.314^{b}	11.344^{f}	31.126^{c}	14.622^{e}
**2**	35.754^{bc}	37.903^{ab}	39.194^{a}	21.654^{e}	34.677^{c}	34.706^{c}	13.034^{f}	28.916^{d}	19.160^{e}
**3**	36.104^{c}	41.927^{a}	39.072^{b}	22.424^{e}	36.17^{c}	36.927^{c}	11.725^{bc}	32.491^{d}	22.950^{e}
**4**	36.344^{bc}	40.410^{a}	37.052^{b}	24.82^{e}	36.698^{b}	32.215^{d}	12.380^{g}	33.528^{cd}	17.106^{f}
**5**	37.536^{a}	38.999^{a}	38.445^{a}	26.172^{d}	34.795^{b}	38.579^{a}	11.398^{f}	31.213^{c}	14.853^{e}
**6**	36.711^{bc}	39.897^{a}	37.18^{b}	22.77^{e}	34.813^{cd}	37.065^{b}	10.360^{g}	33.626^{d}	21.233^{f}
**7**	37.819^{b}	38.228^{b}	42.113^{a}	26.403^{d}	37.112^{b}	37.033^{b}	11.424^{f}	30.074^{c}	18.839^{e}
**8**	36.756^{bc}	38.073^{b}	40.697^{a}	24.080^{e}	35.196^{cd}	34.429^{d}	12.407^{g}	33.385^{d}	17.708^{f}
**9**	37.050^{cd}	38.150^{bc}	39.949^{a}	24.471^{f}	36.031^{d}	39.509^{ab}	9.685^{h}	33.669^{e}	16.959^{g}
**10**	38.631^{bcd}	39.781^{abc}	41.819^{a}	23.580^{e}	37.187^{d}	41.123^{ab}	16.594^{g}	37.632^{cd}	20.28^{f}
