# Supplementary material for: Rise and Fall of Phytophthora infestans Resistance to Non-Specific Fungicide in Experimental Populations
Source: J Fungi (Basel). 2025 Aug 30;11(9):643. doi: 10.3390/jof11090643 (PMC12470341; doi:10.3390/jof11090643)
Supplement: Supplementary file 1 [file jof-11-00643-s001.zip › Supplementary Tables/Table S5.pdf]

Table S5 The 14 candidate genes included in ABC transporters or endocytosis pathway.

Gene ID	KEGG pathway
PInf 002354	ABC transporters (ko02010)
PInf 008878	ABC transporters (ko02010)
PInf 008932	ABC transporters (ko02010)
PInf 019050	ABC transporters (ko02010)
PInf 026568	ABC transporters (ko02010)
PInf 014761	Endocytosis (ko04144)
PInf 019693	Endocytosis (ko04144)
PInf 019670	Endocytosis (ko04144)
PInf 004509	Endocytosis (ko04144)
PInf 023130	Endocytosis (ko04144)
PInf 011614	Endocytosis (ko04144)
PInf 021871	Endocytosis (ko04144)
PInf 019030	Endocytosis (ko04144)
PInf 023024	Endocytosis (ko04144)
