# Supplementary figures and images for: Rise and Fall of Phytophthora infestans Resistance to Non-Specific Fungicide in Experimental Populations
Source: J Fungi (Basel). 2025 Aug 30;11(9):643. doi: 10.3390/jof11090643 (PMC12470341; doi:10.3390/jof11090643)

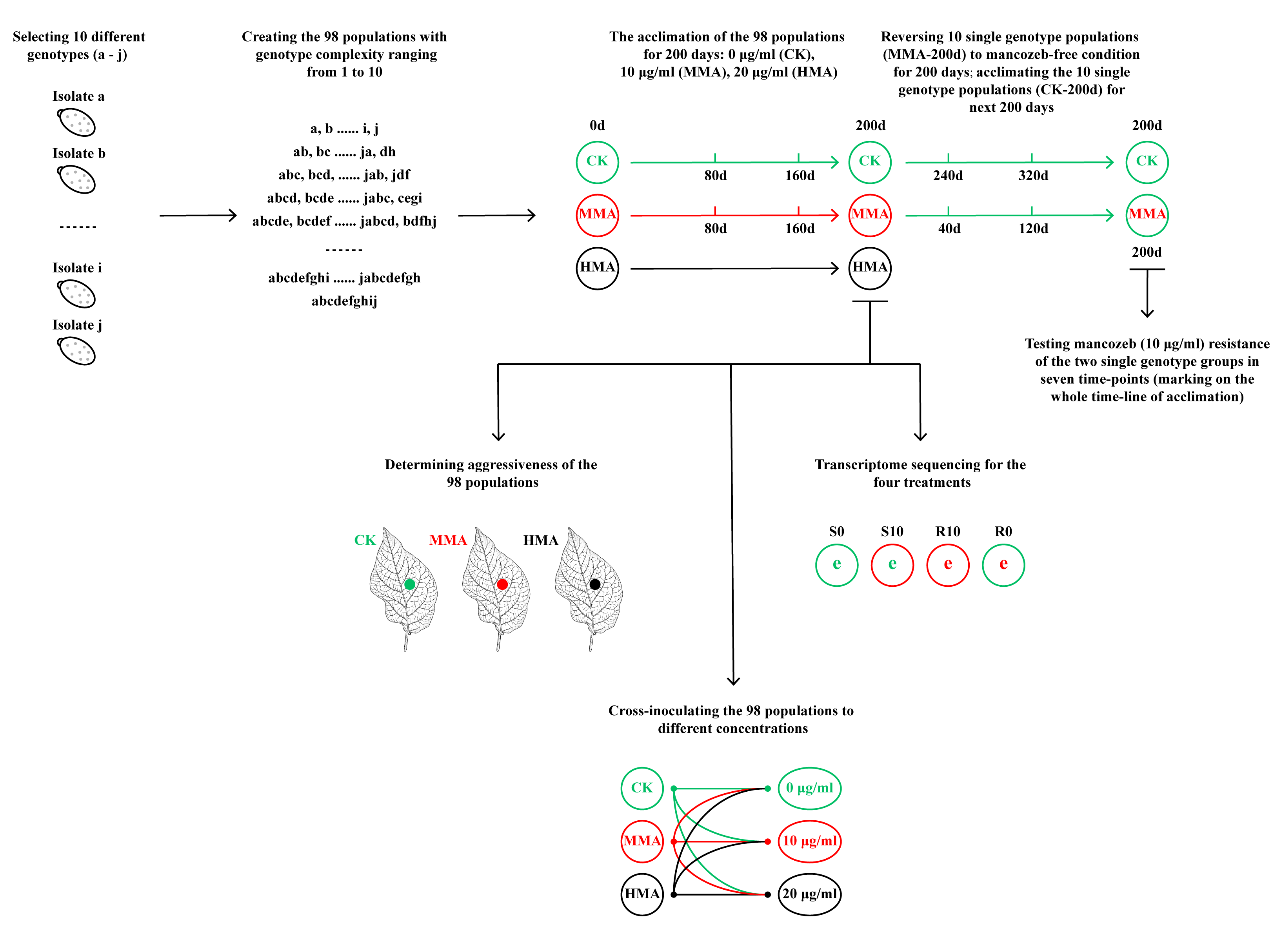

Supplement: Supplementary file 1 [file jof-11-00643-s001.zip › Supplementary Figures/Fig. S1.tif]

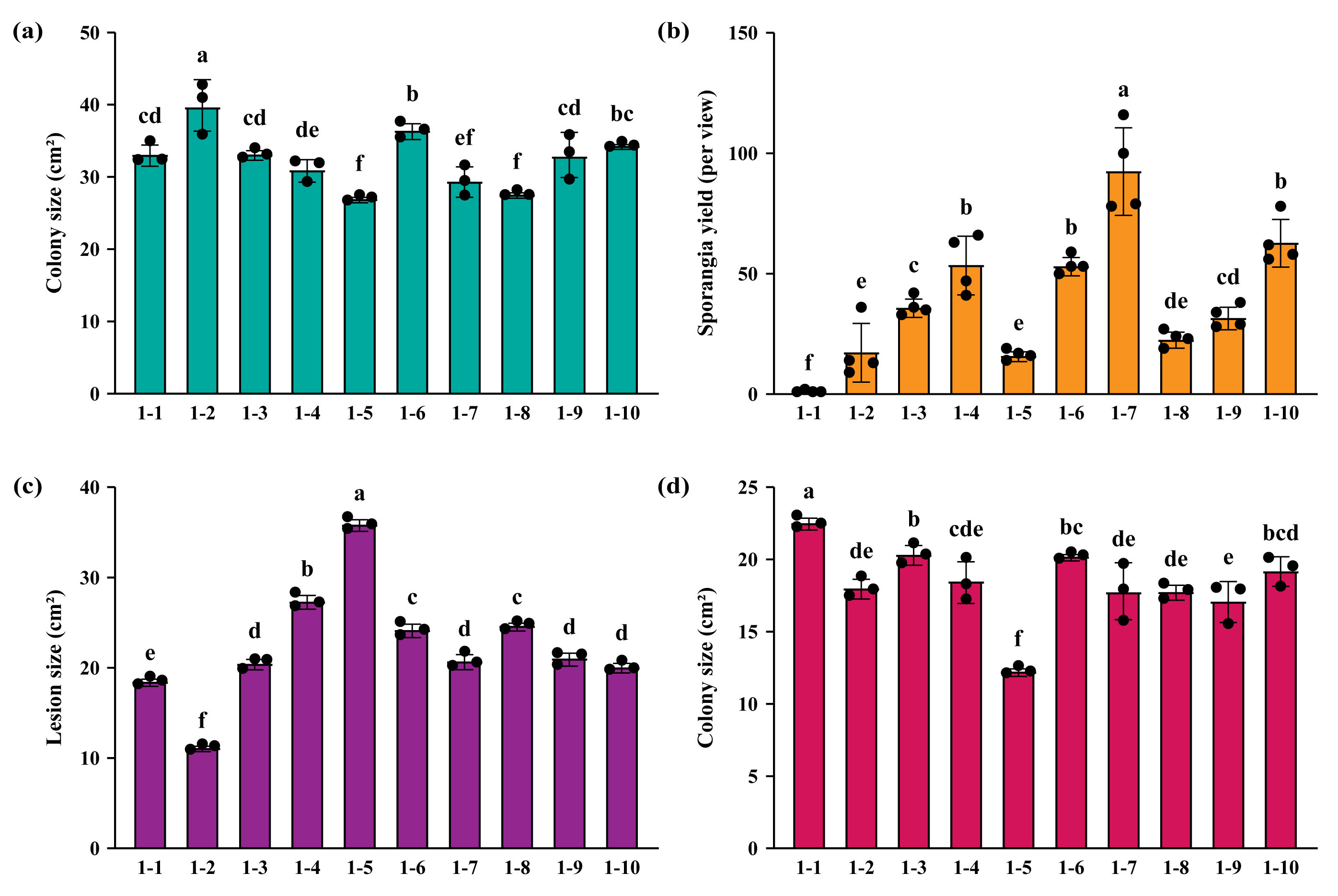

Supplement: Supplementary file 1 [file jof-11-00643-s001.zip › Supplementary Figures/Fig. S2.tif]

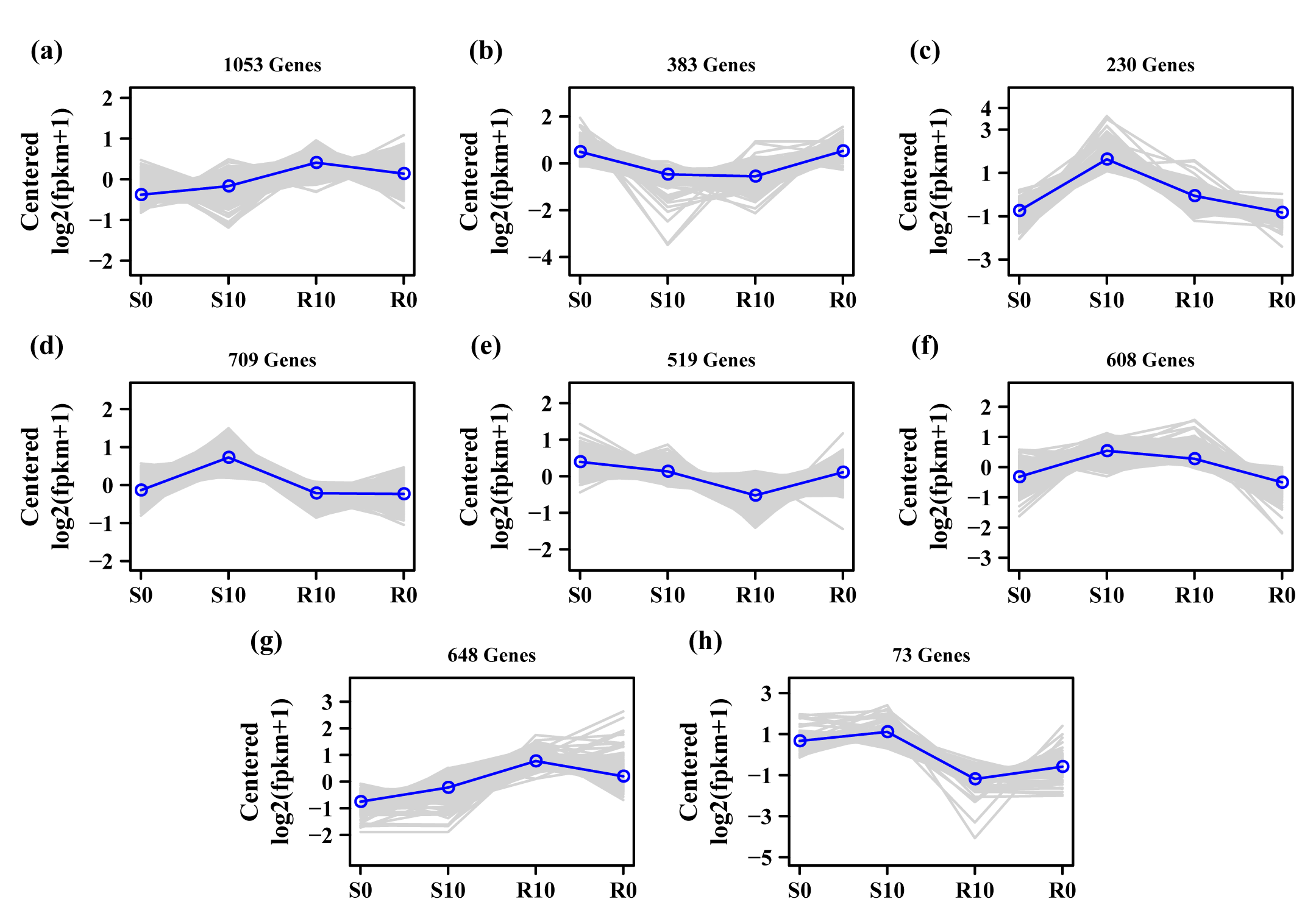

Supplement: Supplementary file 1 [file jof-11-00643-s001.zip › Supplementary Figures/Fig. S3.tif]
